# Pre-pregnancy body mass index classification and weight gain according to new brazilian protocols and their association with gestational diabetes mellitus

**DOI:** 10.61622/rbgo/2026rbgo102

**Published:** 2026-02-20

**Authors:** Lucas Almeida das Chagas, Luiz Gonzaga Ribeiro Silva, Rosângela Maria Lopes de Sousa, Ana Vitória Almeida das Chagas da Silva, Rosy Ane de Jesus Pereira Araújo Barros, Flávio Américo Barroso, Bianca de Almeida Pititto, Edward Araujo Júnior, Rosiane Mattar

**Affiliations:** 1 Universidade Federal de São Paulo Escola Paulista de Medicina Departamento de Obstetrícia São Paulo SP Brazil Departamento de Obstetrícia, Escola Paulista de Medicina, Universidade Federal de São Paulo, São Paulo, SP, Brazil.; 2 Universidade Federal de São Paulo Escola Paulista de Medicina Departamento de Nutrição São Paulo SP Brazil Departamento de Nutrição, Escola Paulista de Medicina, Universidade Federal de São Paulo, São Paulo, SP, Brazil.; 3 Universidade CEUMA Departamento de Nutrição São Luís MA Brazil Departamento de Nutrição, Universidade CEUMA, São Luís, MA, Brazil.; 4 Faculdade Diferencial Integral Departamento de Farmácia Teresina PI Brazil Departamento de Farmácia, Faculdade Diferencial Integral, Teresina, PI, Brazil.; 5 Departamento de Medicina da Universidade Federal do Maranhão São Luiz MA Brazil Departamento de Medicina da Universidade Federal do Maranhão, São Luiz, MA, Brazil.; 6 Departamento de Obstetrícia e Ginecologia da Universidade Federal do Maranhão São Luiz MA Brazil Departamento de Obstetrícia e Ginecologia da Universidade Federal do Maranhão, São Luiz, MA, Brazil.; 7 Universidade Federal de São Paulo Escola Paulista de Medicina Departamento de Medicina Preventiva São Paulo SP Brazil Departamento de Medicina Preventiva, Escola Paulista de Medicina, Universidade Federal de São Paulo, São Paulo, SP, Brazil.

**Keywords:** Body mass index, Gestational diabetes mellitus, Gestational weight gain, Pregnancy, Pregnant people

## Abstract

**Objective:**

This study investigated the association between gestational weight gain based on the new weight gain curve adopted by the Brazilian Ministry of Health for pregnant women and the risk of developing Gestational Diabetes Mellitus (GDM).

**Methods:**

Cross-sectional study was conducted with 104 pregnant women—52 with GDM and 52 without—matched for age, self-reported skin color, pre-existing hypertension, and family history of type 2 diabetes. Statistical analyses were performed using SPSS version 19.0. Categorical variables were analyzed using the Chi-square or Fisher's exact test, and continuous variables with the Student's t-test or Mann–Whitney U test. Two multivariate logistic regression models were applied: one using BMI and GWG as continuous variables and another using their categorical classifications. Odds ratios (OR) were calculated as exp(b), and models were adjusted for age, skin color, income, education, and family history of diabetes. A p-value < 0.05 was considered significant.

**Results:**

Women with GDM reported less physical activity before (26.9% vs. 46.2% p = 0.042) and during pregnancy (17.3% vs. 40.4% p = 0.009). Regarding pre-gestational nutritional status, women with GDM showed a higher prevalence of overweight (53.9% vs. 30.8%), while obesity was more frequent among women without GDM (40.4% vs. 26.9%), although these differences were not statistically significant (p = 0.059). Women with GDM had lower median gestational weight gain (4.5 kg vs. 8.0 kg, p < 0.001) and a higher proportion exceeded the recommended values (84.6% vs. 40.4%). Excessive GWG was significantly associated with GDM: each additional kilogram gained increased the odds of developing GDM by 28% (aOR 1.28; 95% CI: 1.12–1.47, p < 0.001).

**Conclusions:**

Excessive gestational weight gain was significantly associated with GDM, underscoring the importance of monitoring maternal weight gain to prevent complications.

## Introduction

Pregnancy is characterized as a period in a woman's life marked by various bodily changes, including gestational weight gain (GWG), to support fetal development and growth.^(1)^ Monitoring GWG is considered an essential part of prenatal care, which should be conducted by a multidisciplinary healthcare team to prevent excessive or insufficient weight gain and associated complications.^(2)^

The prevalence of overweight and obesity among women of childbearing age has been increasing in recent years.^(2)^ Evidence shows that women with pre-pregnancy body mass index (BMI) in the overweight or obese categories and those with excessive GWG are more likely to experience adverse perinatal outcomes such as preterm birth, hypertension, fetal macrosomia, and an increased risk of developing gestational diabetes mellitus (GDM).^(3,4)^ GDM is characterized by varying degrees of carbohydrate intolerance that can arise during pregnancy, making it one of the most common complications of pregnancy.^(5)^ The global prevalence of this condition ranges from 8.4% to 30.9% and is associated with population characteristics such as age and obesity, as well as the different diagnostic methods used.^(6,7)^

In recent years, much discussion has centered on the consequences of inadequate pre-pregnancy and gestational nutritional status on pregnancy quality and potential complications for both mother and offspring.^(4,8,9)^ Pregnant women who are overweight or obese face twice the risk of developing GDM compared to non-obese women.^(10,11)^ On the other hand, underweight during pregnancy raises concerns about fetal development and the risk of prematurity and mortality.^(8)^ These complications arising from poor maternal nutritional status pose concerns for healthcare systems worldwide.^(12)^

Pre-pregnancy BMI is considered a strong predictor of weight gain during pregnancy.^(1,13)^ According to the guidelines of the Institute of Medicine (IOM), the recommended total weight gain for underweight pregnant women is 12.5–18 kg, for normal-weight women is 11.5–16 kg, for overweight women is 7–11.5 kg, and for obese women is 5–9 kg.^(1)^ However, pregnant women often exceed these recommended GWG targets, increasing the risk of maternal and fetal complications.^(11)^

Historically, Brazil did not have its own nutritional recommendations for GWG, relying instead on guidelines from other countries.^(1,14-17)^ Until 2022, the country used the IOM recommendations and Atalah's et al. chart for GWG classification.^(1,17)^ However, based on data from the Brazilian Maternal-Child Nutrition Consortium (CONMAI) in 2021, which aimed to investigate the health of the general population and maternal-child health,^(18)^ the Brazilian Ministry of Health (MoH) approved new GWG curves for the Brazilian population, which are currently in use for the classification of pre-pregnancy status and GWG estimates.^(19)^

To prevent potential errors arising from the nutritional classification of pregnant women and the higher risk of adverse perinatal outcomes associated with overweight and/or obesity during pregnancy, the MoH recommends that pregnant women be monitored using this newly validated GWG curve.^(19)^

Based on the new GWG classification validated in Brazil in 2022, this study aimed to assess the pre-pregnancy nutritional status and evaluate GWG, using the tool approved by the MoH, in women with and without a GDM diagnosis.

## Methods

Cross-sectional study comparing pre-gestational BMI and GWG in women with and without GDM according to the new criteria proposed by the BHM. This study was conducted by the Paulista School of Medicine - Federal University of São Paulo (EPM-UNIFESP) with data collection in the city of São Luís, Maranhão, Northeast of Brazil. Data collection took place between December 2023 and June 2024.

All pregnant women attended at the Outpatient Units in the city of São Luís, received prenatal care and underwent fasting blood glucose testing in the first trimester as part of the routine outpatient protocol. Those with normal fasting blood glucose (< 92 mg/dL) were subjected to the 75g 2-hour oral glucose tolerance test (OGTT) between 24-32 weeks of gestation. Pregnant women who met any of the following criteria were diagnosed with GDM: fasting glucose 92-125 mg/dL, 1-hour glucose >180 mg/dL, 2-hour glucose 153-199 mg/dL.^(5)^ All pregnant women diagnosed with GDM within the municipal or state health network were referred to the Diabetes Outpatient Clinic of the Maternal and Child University Hospital or the High Complexity Maternity Outpatient Clinic, which are public outpatient clinics where these women continued their prenatal care with a specialized multidisciplinary health team.

In both outpatient clinics, data for the study were collected during the first consultation, before the patients whether diagnosed with GDM or not had any contact with other healthcare professionals. The responsible researcher (a nutritionist) invited them to participate in the study. Inclusion criteria for participation considered women aged 18-45 years, having completed the OGTT and presenting the results at the time of recruitment, gestational age between the 24th and 32nd weeks (based on an ultrasound performed before the 20th week), singleton pregnancy with a live fetus, and no prior nutritional guidance on healthy eating for GDM control.

Pregnant women were deemed ineligible if they did not present the OGTT results at recruitment, were foreigners, or lacked proficiency in the Portuguese language. Other exclusion criteria included mental, auditory, or visual impairments that could hinder data collection, history of solid organ transplants, pre-gestational hyperglycemia (type 1 or type 2 diabetes mellitus), previous bariatric surgery, history of food intolerance (to lactose, gluten, or others) or food allergies, use of corticosteroids, diuretics, or other medications affecting blood glucose or appetite (such as prednisone, dexamethasone, furosemide, bumetanide, among others), and those with hyperemesis.

All eligible women responded to an oral questionnaire about sociodemographic and clinical history information. This included age, self-reported skin color, marital status, educational level, family income, religion, family history of diabetes, history of hypertension, alcohol and tobacco use before pregnancy, pre-gestational weight, current weight for GWG classification, and regular physical activity (intensity, frequency per week, and duration in minutes) in the six months prior to pregnancy. Pregnant women who reported engaging in physical activity exceeding 30 minutes per session and more than 3-4 times per week were considered physically active.^(20)^

After collecting sociodemographic and clinical data, information on the participants’ self-reported pre-gestational weight and recorded height (in meters) was gathered to calculate and classify their pre-gestational BMI as underweight (<18.5 kg/m^2^), normal weight (18.5-24.9 kg/m^2^), overweight (25.0-29.9 kg/m^2^), or obesity (≥30.0 kg/m^2^).^(1)^ For the GWG calculation, the gestational weight at the time of recruitment was subtracted from the reported pre-gestational weight. GWG was then standardized for gestational age in order to obtain z-scores and percentiles specific to each participant's pre-gestational BMI, according to the Brazilian GWG chart. Finally, weight gain was classified as below, within, or above the recommendations.^(19)^

The sampling for this study was by convenience, with a total of 104 participants who met the eligibility criteria, consisting of 52 participants diagnosed with GDM and 52 without the diagnosis. The groups were matched for age (± 2 years), self-reported skin color (Black, White, and Mixed-race), pre-existing hypertension (yes or no), and family history of type 2 diabetes mellitus (yes or no).

For this study, we divided the 104 participants into two groups based on the presence or absence of a GDM diagnosis during pregnancy. The first group (G1) consisted only of women diagnosed with GDM, while the second group (G2) included all those without a GDM diagnosis.

The SPSS software (IBM version 19.0, Armonk, NY, USA) was used for the analysis of all variables. The values are expressed as numbers (percentages), mean (standard deviation) or median (interquartile range). The Chi-square test was used to classify the pregnant women according to categorical variables related to sociodemographic and clinical characteristics. The Chi-square test or Fisher's exact test were used to compare the participants’ characteristics regarding pre-gestational BMI and GWG.

The variance-inflation Factor (VIF) performed the multicollinearity test, adopting < 5 as the perceived absence of multicollinearity as the cut-off point. Two multivariate logistic regression analyses were conducted. In Regression 1, the BMI and gestational weight gain (GWG) variables were included as continuous variables. In Regression 2, categorical classifications of BMI and GWG were used.

For the calculation of the odds ratio (OR) in Regression 1, the continuous variables (pre-gestational BMI in kg/m^2^ and GWG in kg) were directly included in the binary logistic regression. The OR value was considered as the exponential of the coefficient b (exp(b)), reflecting the increased likelihood of the outcome occurring for each unit increase in the respective variable. Thus, the OR for GWG represents the increased probability of developing gestational diabetes mellitus (GDM) for each additional kilogram gained during pregnancy. Similarly, the OR for BMI reflects the increased probability of GDM for each 1 kg/m^2^ increment in pre-gestational BMI. Both regressions presented crude and adjusted results. Adjustments were made based on the following covariates: age, skin color, income, education level, and family history of diabetes. p-values < 0.05 were considered statistically significant.

This study was approved by the Research Ethics Committee of the Federal University of São Paulo (UNIFESP) under protocol number CAAE 72720723.0.0000.5505and opinion number 6.569.425. All participants were informed about the study objectives and procedures and provided written informed consent prior to inclusion.

## Results

Our findings demonstrate that pregnant women with GDM reported engaging in less physical activity before pregnancy (26.9% vs. 46.2%, p < 0.042) and during pregnancy compared to those without GDM (17.3% vs. 40.4%, p < 0.009). The other characteristics of the participants did not differ significantly between the two groups (Table 1).

**Table 1:** Sociodemographic and clinical characteristics of the 104 pregnant women with and without gestational diabetes mellitus

Variables		Diagnosis of GDM		p-value
		G1(n = 52) n(%)	G2(n = 52) n(%)	
Age, years, Mean±SD		31.5±6.3	31.2±6.8	0.834 ¥
Gestational age, weeks, Mean (SD)		27.5±3.2	27.8±3.1	0.577 ¥
Race				
	Mixed-race	29(55.8)	31(59.6)	0.916 §
	White	7(13.5)	6(11.5)	
	Black	16(30.8)	15(28.8)	
Marital status				
	Married	14(26.9)	21(40.4)	0.146 §
	Single	38(73.1)	31(59.6)	
Education				
	0-8 years	7(13.5)	3 (5.8)	0.183 §
	≥ 8 years	45(86.5)	49 (94.2)	
Monthly family income				
	< 2 minimum wages	25(48.1)	30(57.7)	0.337 §
	≥ 2 minimum wages	27(51.9)	22 (42.3)	
Religion				
	Catholic	14(26.9)	22(42.3)	0.099 §
	Others	38(73.1)	30(57.7)	
Smoking				
	No	49(94.2)	47(90.4)	0.462 §
	Yes	3(5.8)	5(9.6)	
Alcohol consumption				
	No	33(63.5)	33(63.5)	1.000 §
	Yes	19(36.5)	19(36.5)	
Family history of diabetes				
	No	27(51,6)	32(61.6)	0.678 §
	Yes	25(48.7)	20(38.4)	
Chronic hypertension				
	No	44(84.6)	45(86.5)	0.780 §
	Yes	8(15.4)	7(13.5)	
Physical activity pre pregnancy				
	No	38(73.1)	28(53.8)	0.042 §
	Yes	14(26.9)	24(46.2)	
Physical activity in pregnancy				
	No	43(82.7)	31(59.6)	0.009 §
	Yes	9(17.3)	21(40.4)	

GDM - Gestational diabetes mellitus; G1 - GDM; G2 - No GDM; Mean±SD - Mean±Standard deviation;

¥ Student's t-test;

§ Chi-squared test

Table 2 presents the classification of pre-gestational BMI and GWG according to the recently adopted Brazilian classification. When assessing pre-gestational nutritional status, women with GDM showed a higher rate of overweight (53.9% vs. 30.8%), while obesity was more frequent among women without GDM (40.4% vs. 26.9%), although the differences did not reach statistical significance (p = 0.059). Regarding GWG, women with GDM had lower median weight gain (4.5 kg vs. 8.0 kg, p < 0.001), and a greater proportion exceeded the recommended values (84.6% vs. 40.4%).

**Table 2:** Evaluation of pre-pregnancy body mass index and gestational weight gain of the 104 pregnant women with and without gestational diabetes mellitus according to Brazilian classification

Variables		Diagnosis of GDM		p-value
		G1 (n = 52) n(%)	G2 (n = 52) n(%)	
Pre-pregnancy BMI, kg/m^2^, Med (IQR)		27.8(7.1)	28.0(4.8)	0.526 ¥
Pre-pregnancy BMI, kg/m^2^, n (%)				
	< 18.5	0(0.0)	0(0.0)	0.059 ¥
	18.5-24.9	10(19.2)	15(28.8)	
	25.0-29.9	28(53.9)	16(30.8)	
	> 30.0	14(26.9)	21(40.4)	
GWG, Med (IQR)		4.5(4.0)	8.0(7.0)	<0.001 ¥
Recommended GWG				
	Below	1(1.9)	3(5.8)	<0.001 ¥
	Within	7(15.5)	28(53.8)	
	Above	44(84.6)	21(40.4)	

GDM - Gestational diabetes mellitus; G1 - GDM; G2 - No GDM; BMI - Body mass Index; GWG - Gestational weight gain; Med (IQR) - Median (Interquartile range);

¥ Mann-Whitney U Test

Table 3 shows the risk of having GDM according to pre-gestational BMI and GWG, as well as their classifications. In Regression 1 greater GWG was associated with increased odds of developing GDM. In the crude analysis, each additional kilogram gained during pregnancy was associated with a 26% higher likelihood of GDM (OR: 1.26; 95% CI: 1.11–1.43, p < 0.001). In the adjusted model, this effect remained significant, with a 28% higher likelihood per kilogram gained (aOR: 1.28; 95% CI: 1.12–1.47, p < 0.001). These results should, however, be interpreted with caution, as Regression 1 did not account for pre-gestational BMI categories, which are known to influence the relationship between GWG and GDM.

**Table 3:** Probability between gestational weight gain and its relationship with the risk of women developing gestational diabetes mellitus

Variables		Raw analysis			Adjusted multivariable*		
		aOR	CI 95%	p-value	aOR	CI 95%	p-value
Regression 1							
Pre-pregnancy BMI		1.05	0.95-1.16	0.302	1.04	0.94-1.15	0.478
GWG		1.26	1.11-1.43	<0.001	1.28	1.12-1.47	<0.001
Regression 2							
Pre-pregnancy BMI, kg/m^2^							
	18.5-24.9	1.00			1.00		
	25.0-29.9	2,63	0.96-7.20	0.061	1.74	0.54-5.65	0.355
	> 30.0	1.01	0.35-2.85	1.000	0.88	0.25-3.07	0.840
Recommended GWG							
	Below	1.00			1.00		
	Within	0.75	0.07-8.35	0.815	0.87	0.07-10.52	0.915
	Above	6.29	0.61-64.10	0.121	6.99	0.60-81.59	0.121

GWG - Gestational weight gain; OR: odds ratio. aOR - adjusted OR (adjusted for age, race, income, education, and family history of diabetes mellitus); CI - confidence interval

## Discussion

In this study, we observed that women diagnosed with GDM were more likely to present inadequate gestational weight gain (GWG) and reported less physical activity before and during pregnancy. Although higher proportions of overweight and obesity were found among women with GDM, these differences did not reach statistical significance in our sample. Nevertheless, these findings highlight the relevance of GWG and lifestyle factors, such as physical activity, as potential contributors to the development of GDM, which is consistent with evidence from the literature.^(10,21-24)^

Maternal obesity is considered a public health problem worldwide.^(12)^ Obese women have a higher risk of developing GDM and greater risk of maternal-fetal complications.^(25)^ In recent years, various studies have been conducted to investigate the effect of "diabesity",^(21)^ which is the relationship between diabetes and pre-pregnancy obesity on perinatal outcomes. Researchers have discovered a strong association between maternal obesity and an increased risk of developing GDM and maternal-fetal complications.^(10,21,22)^ These investigations support our findings. In our study, we observed that pregnant women with GDM tended to have higher pre-gestational BMI, indicating overweight and obesity, compared to those without GDM. However, these differences were not statistically significant in our sample, which may be explained by the limited sample size. Even so, the literature consistently shows that maternal overweight and obesity are important risk factors for GDM and adverse perinatal outcomes.^(10,21,22,25)^

Additionally, through the GWG curve, we noted that pregnant women in the GDM group exhibited excessive weight gain for the gestational age percentile, whereas the control group had weight gain within the recommended range. This suggests that the risk of GDM in our sample was more strongly associated with excessive GWG than with pre-gestational BMI, which aligns with previous studies showing GWG as an independent predictor of adverse outcomes.^(19,25,26)^

Currently, BMI is used to classify the nutritional status of the general population and as an instrument to estimate GWG. Excessive GWG not only increases the risk of GDM but is also associated with the development of gestational hypertensive disease and a higher incidence of cesarean sections.^(24,25)^ Overweight and obesity in pregnant women lead to greater fat accumulation in the pelvic cavity, resulting in reduced pelvic space and obstructing fetal head descent, prolonging labor and increasing the risk of complications such as postpartum hemorrhages.^(27)^ However, adopting lifestyle changes, including regular physical activity, can reduce the risk of these complications.^(23)^

Regular physical activity before and during pregnancy is an important factor that helps in maintaining adequate GWG, reducing the risk of GDM and maternal-fetal complications by aiding in glucose regulation.^(28)^ According to findings by Xie et al.,^(23)^ decreased physical activity during pregnancy is associated with increased excessive weight gain and a higher risk of pregnant women developing GDM and other maternal-fetal complications arising from the absence of physical activity.^(23)^ These results align with our findings that women with GDM reported less physical activity, which was associated with higher GWG compared to women without GDM. Our findings are also consistent with meta-analyses by Tobias et al.^(24)^ and Mijatovic-Vukas et al.,^(29)^ both of which highlighted the relationship between reduced physical activity and a higher risk of GDM.

Therefore, GWG is considered a critical marker that defines various nutritional and physiological conditions during pregnancy, and regular physical activity can help reduce these complications. Several studies suggest that GWG in obese pregnant women should be controlled to prevent adverse pregnancy outcomes resulting from excessive GWG. These studies recommend adhering to the current GWG guidelines proposed by the IOM. In Brazil, it is currently recommended to follow local guidelines to prevent such complications.

We acknowledge that this study has several limitations. The small sample size may have been insufficient. Additionally, the lack of dietary surveys assessing the relationship between excessive GWG and diet, given the important role of nutrition in defining nutritional status, is a limitation. Furthermore, the use of self-reported pre-gestational weight may have introduced recall bias. Another important point is that the regression analysis did not fully adjust for pre-pregnancy BMI categories, a well-recognized strong and independent factor in determining the risk of GDM. This methodological limitation reduces the model's ability to isolate the true effect of GWG, so the results should be interpreted with caution. Future studies should apply statistical models that simultaneously account for pre-pregnancy BMI and GWG in order to better clarify whether GWG acts independently or as a mediator of the relationship between prior obesity and GDM.

## Conclusion

This is the first study to apply the new gestational weight gain curve for Brazilian pregnant women to investigate its association with GDM. Excessive gestational weight gain was identified as a significant risk factor, while pre-pregnancy BMI showed only a non-significant trend. Given the methodological limitation of not fully adjusting for BMI categories, the results should be interpreted with caution. Further studies with larger and more diverse samples are needed to validate these findings and strengthen the use of this new tool in clinical practice.

## Data Availability

The authors did not make the data from this article available in repositories prior to submission.
